# Patient Selection and Clinical Indication for Chronic Total Occlusion Revascularization—A Workflow Focusing on Non-Invasive Cardiac Imaging

**DOI:** 10.3390/life13010004

**Published:** 2022-12-20

**Authors:** Kevin Hamzaraj, Andreas Kammerlander, Mariann Gyöngyösi, Bernhard Frey, Klaus Distelmaier, Senta Graf

**Affiliations:** Department of Internal Medicine II, Division of Cardiology, Medical University of Vienna, 1090 Vienna, Austria

**Keywords:** chronic total occlusion, cardiac magnetic resonance, echocardiography, positron emission tomography, single photon emission computer tomography, coronary artery disease

## Abstract

Percutaneous coronary intervention of chronic total occlusion (CTO PCI) is a challenging procedure with high complication rates and, as not yet fully understood long-term clinical benefits. Ischemic symptom relief in patients with high ischemic burden is to date the only established clinical indication to undergo CTO PCI, supported by randomized controlled trials. In this context, current guidelines suggest attempting CTO PCI only in non-invasively assessed viable CTO correspondent myocardial territories, with large ischemic areas. Hence, besides a comprehensive coronary angiography lesion evaluation, the information derived from non-invasive cardiac imaging techniques is crucial to selecting candidates who may benefit from the revascularization of the occluded vessel. Currently, there are no clear recommendations for a non-invasive myocardial evaluation or choice of imaging modality pre-CTO PCI. Therefore, selecting among available options is left to the physician’s discretion. As CTO PCI is strongly recommended to be carried out explicitly in experienced centers, full access to non-invasive imaging for risk-benefit assessment as well as a systematic institutional evaluation process has to be encouraged. In this framework, we opted to review the current myocardial imaging tools and their use for indicating a CTO PCI. Furthermore, based on our experience, we propose a cost-effective systematic approach for myocardial assessment to help guide clinical decision-making for patients presenting with chronic total occlusions.

## 1. Introduction to Chronic Total Occlusion and Revascularization Recommendations

Chronic total occlusion (CTO) of coronary arteries represents an advanced form of atherosclerotic coronary artery disease, which is currently prevalent in circa one-fifth of patients presenting for diagnostic coronary angiography [1]. CTO is defined as a chronic occlusion of the artery for longer than 3 months with a TIMI 0 flow and is associated with the development of collateral conduits from donor vessels that maintain a certain perfusion level to the CTO-related myocardial segments [2,3]. However, these collaterals are very often insufficient to provide adequate myocardial perfusion, which often leads to the typical manifestation of ischemic heart disease [2]. A growing body of evidence suggests that the revascularization of CTO using coronary artery bypass grafting or percutaneous coronary intervention (PCI) has several clinical benefits, including ischemic symptom relief and quality of life improvement. These findings are supported to date by limited randomized controlled studies assessing the effects of CTO PCI upon clinical indication [4,5,6,7] (Table 1). Yet it is still unclear whether revascularization of CTO provides a survival benefit or long-term freedom from cardiac events, compared to receiving optimal medical therapy alone–indeed, the few available randomized controlled trials have reported no benefit in this context [5,7]. However, large observational studies on CTO patients have concordantly been reporting positive effects of CTO PCI on long-term survival and freedom from cardiac events. Of note, most of these studies compared patients that underwent successful vs. unsuccessful revascularization attempts on CTO vessels [8,9,10].

Due to the lack of randomized trials, the hard-outcome benefits of CTO PCI are not yet fully elaborated. However, the HORIZONS-AMI trial observed that the presence of a CTO in patients undergoing PCI for ST-elevation myocardial infarction was associated with worse early and late clinical outcomes [11].

CTO PCI is a challenging procedure with increased technical complexity and a need for appropriate operator experience. Through recent advancements and the development of dedicated methods and devices in the last few years, success rates of CTO PCI have increased significantly. However, they remain lower than non-CTO PCI–#, with successful revascularization of CTO vessels ranging from 60–70% of patients in inexperienced operating centers and 90–95% in some highly experienced centers [10,12,13,14]. Moreover, these patients are characterized by a higher interventional complication risk compared to non-CTO PCI, suggesting the need for a careful patient selection and benefit-risk evaluation before attempting CTO PCI, adapted to operator experience and expected symptom and prognostic benefits [3,15,16].

The European Society of Cardiology (ESC) guidelines on myocardial revascularization suggest choosing patients for CTO PCI in a similar manner to those who need treatment for non-CTO lesions, and explain that clinical benefits are analogous among these patient groups–hence, the rationale and criteria for decision-making in the revascularization of stable CAD should apply to the CTO subset [16]. As stated in the guidelines, prognostic benefits of revascularization may be granted to patients with a significant left main and/or left anterior descending artery (LAD) stenosis, multi-vessel disease or in patients with an ischemic territory exceeding 10% of the left ventricle. For this reason, they suggest an objective quantification of ischemia using non-invasive diagnostic imaging as a first-line test before revascularization. In left ventricular dysfunction, guidelines recommend viability testing to be performed appropriately for the detection of stunned or hibernating myocardium causing heart failure with the potential of functional recovery [16].

Most importantly, CTO PCI is currently recommended by the ESC in selected patients with angina symptoms resistant to medical therapy (class of recommendation II-a, level of evidence B) [16] (Table 2). However, the 2017 American College of Cardiology/American Heart Association guidelines on myocardial revascularization recommend CTO PCI only upon clinical indication and in the hands of appropriately experienced operators, as a class II-a of recommendation and level of evidence B [15]. The recent 2021 American guidelines downgraded the clinical recommendation for CTO PCI to a class II-b level of evidence B due to equivocal evidence based on randomized trials: “In patients with suitable anatomy who have refractory angina on medical therapy, after treatment of non-CTO lesions, the benefit of PCI of a CTO to improve symptoms is uncertain”. They also encourage CTO PCI after shared-decision and potential benefits [17] (Table 2). Of note, no randomized trials comparing CTO PCI and CABG are available to date.

Pre-interventional evaluation of CTO lesions has indeed to be well elaborated as the main characteristics of this specific lesion subset, such as collateral vessels and complete antegrade flow impairment, restrict diagnostic availability or alter the interpretations for clinical indication. For example, the use of the broadly recommended FFR or the novel CT-FFR measurements is not routinely possible in CTO vessels [16]. Thus, non-invasive imaging takes on greater significance and the choice of techniques and interpretation of imaging-derived information require special attention.

In this context, suitable candidates to undergo CTO PCI should be carefully identified and selected taking into consideration diverse clinical factors and supported by appropriate cardiac imaging techniques evaluating viability and ischemia.

Guided by current recommendations and clinical practice, we opted to review the available evidence on benefits of CTO PCI and shed light on pre-interventional requirements for the consideration of revascularization. Throughout our review, we used the term revascularization to reference PCI as our primary focus, if not stated otherwise. Furthermore, we summarised the available non-invasive imaging methods that support the physician to guide the patient selection process.

## 2. Which Patient May Benefit from CTO PCI?

### 2.1. Viability

In patients with coronary artery disease and normal ventricular function without regional wall motion abnormalities assessed by echocardiography, intact myocardial viability can be presumed [18]. In these patients, several benefits of revascularization have been reported. In patients with preserved systolic left ventricular function and one single vessel disease randomized in the ORBITA trial, revascularization improved the stress wall motion score index as assessed by cardiac echocardiography after 6 weeks as a secondary endpoint [19]. Furthermore, a large meta-analysis described better outcomes of revascularization in patients with viable myocardium and normal left ventricular function, as compared to medical therapy [20].

On the other hand, the clinical benefit of revascularization in patients with left ventricular dysfunction is still ambivalent. A considerable proportion of CTO patients manifest heart failure with a reduction of left ventricular function [10] but it is unclear still whether CTO PCI is able to induce recovery. One large randomized controlled trial (*n* = 205) investigated the left ventricular recovery in terms of wall thickness and ejection fraction and found no differences between CTO patients who underwent revascularization and those who received optimal medical therapy alone. However, the results were limited by the low rates of ventricular dysfunction at baseline and the revascularization of diseased donor vessels in the control group [6]. However, previous studies have reported positive results in the general CAD population. A large meta-analysis of 3088 patients studied the role of myocardial viability in the revascularization of CAD patients with severe left ventricular dysfunction (as assessed by the left ventricular ejection fraction) [21]. This study underlined that viable myocardium benefits immensely from revascularization as compared to medical therapy and paved the way for further research and clinical applications. Its implications may have a slightly different meaning nowadays, as, during the few past years, medical therapy for heart failure has witnessed massive improvements; patients treated medically in the current era have a better prognosis with the new heart failure therapies, as reported in large randomized controlled studies [22,23]. However, the interpretation for clinical practice was limited by the observational nature of the study and the lack of information on the method of revascularization. Later on, most solid data came from randomized trials on patients receiving coronary artery bypass grafting (CABG), suggesting that ischemic but viable myocardium with left ventricular dysfunction has a better long-term prognosis after CABG [24]. A viability sub-study of the STICH trial on patients with reduced left ventricular function receiving CABG reported at first less cardiac mortality and cardiac hospitalization within 5 years when myocardial viability was preserved. However, in the multivariable analysis, the correlation was lost [25]. On the other hand, in an extended 10-year follow-up, freedom from cardiac death and hospitalization was significantly higher in the STICH trial patients when myocardial viability was preserved [26].

It seems that revascularization in ventricular dysfunction has prognostic benefits, but it has been long debated if this implication depends on the revascularization method. Indeed, in the general CAD population, the recent FAME-3 trial reported a non-inferiority of functionally-guided PCI vs. CABG in 1-year follow-up. However, patients with left ventricular dysfunction were underrepresented with ca. 18% in both treatment arms [27].

Recently, one randomized trial recently addressed the evidence gap. The REVIVED trial investigated patients with viable dysfunctional left ventricles undergoing PCI and reported no benefit in survival or cardiac events in 3 years compared to the control group which received optimal medical therapy alone. Moreover, the trial showed no improvement in left ventricular ejection fraction after PCI [28]. However, the clinical endpoint observation time might have been too early in the REVIVED trial: As seen in the STICH trial, prognostic benefits of revascularization may be detected only after a longer observational period. Another issue might be the non-adequate selection of patients with left ventricular dysfunction for myocardial revascularization. In the PARR-2 randomized trial, patients identified using PET before undergoing PCI had better hard outcomes than those selected using the standard of care protocol [29]. Despite studying a smaller cohort than REVIVED, this study emphasizes the need for a more careful clinical indication by highly sensitive methods of non-invasive cardiac imaging.

On the other hand, non-randomized data suggest PCI survival benefits in left ventricular dysfunction: Gerber et al., reported a higher 3-year survival in patients with severe left ventricular dysfunction and viable myocardium who received revascularization. Overall, the study reported similar 3-year mortality as the REVIVED trial [30]. Although emerging data may lead to discussions in the next guidelines, current practices and indications for patients with myocardial dysfunction undergoing revascularization (including CTO patients) will most probably remain unaltered [31].

In patients with ventricular dysfunction, PCI benefits may be found mostly in the presence of hibernation. An observational study on 648 patients reported that an extent of hibernating myocardium exceeding 10% was associated with the benefits of revascularization [32]. Physiologically, improvement in left ventricular function may be physiologically explained by reversed myocardial hibernation after restored perfusion, with enhanced reversibility in those patients who have less fibrotic tissue [33,34]. When left untreated, hibernation can be a progressive condition with subsequent development of fibrosis, myocardial thinning and akinesia [35]. A prospective trial found progressive loss of myocardial viability in patients with ventricular dysfunction receiving neither revascularization nor medical treatment, resulting in scar formation in former hibernating myocardial segments [36]. Of note, revascularization of hibernating myocardium has been associated with improved long-term prognosis in viable areas larger than 10% of the left ventricle [32]. As such, quantification of viability has prognostic value, but is only possible non-invasively.

### 2.2. Ischemia

Revascularization of ischemic but viable myocardium aims to minimize residual ischemia and subsequently improves symptoms and prognosis. Patients with a large ischemic burden (more than 10%) are considered to benefit the most from PCI [37]. This statement is supported mainly by the randomized COURAGE trial, which reported a survival benefit and reduced myocardial infarction rates in patients with an ischemic burden of more than 10% at the baseline and less than 5% after revascularization [38]. In this study, ischemia was evaluated non-invasively using SPECT. On the other hand, a sub-study of the PARR-2 trial using PET reported fewer cardiac events after revascularization in CAD patients with an ischemic but viable myocardial area of more than 7% of the left ventricle [39]. However, the threshold of ischemia in 10% of the myocardium remains standard of care, as this amount of ischemic burden is associated with prognostic benefits of revascularization in the general CAD population [16].

Nowadays, invasive functional assessment of coronary artery stenoses can derive information related to the extent of ischemia in the distal supply region. Treatment of functionally significant stenoses, as assessed by fractional flow reserve, has been proven to be superior to revascularization guided by anatomical evaluation alone. The FAME trial reported better 2-year MACE rates after revascularization of ischemic myocardium, as assessed invasively with FFR [40]. However, for quantitative measurement of myocardial ischemia, coronary flow reserve using PET represents the most reliable parameter due to the detection of ischemia in the whole myocardium, which surpasses the invasive tool of FFR measuring the pressure drop solely [41]. In fact, invasive functional measurement does not apply to CTO lesions, as collateral vessels rather than CTO vessels themselves supply the corresponding myocardial regions.

Indeed, CTO-related myocardium can be an ischemic area even in well-developed collaterals. Werner et al., reported a sufficient collateral flow in only 5% of CTO patients with preserved left ventricular function [42]. When patients report typical symptoms, ischemia is mostly present. A quantitative correlation between ischemic burden and clinical benefits in CTO patients is not specifically stated. The IMPACTOR-CTO trial aimed to stratify patients according to their ischemic burden, guided by the belief that large ischemic CTO-related areas will benefit most from revascularization [43]. This was the only randomized study to report a significant myocardial ischemia reduction in patients undergoing CTO PCI. However, myocardial ischemia reduction remains the primary benefit of CTO PCI.

## 3. Non-Invasive Tools for Assessment of Viability and Ischemia

**Figure 1:**
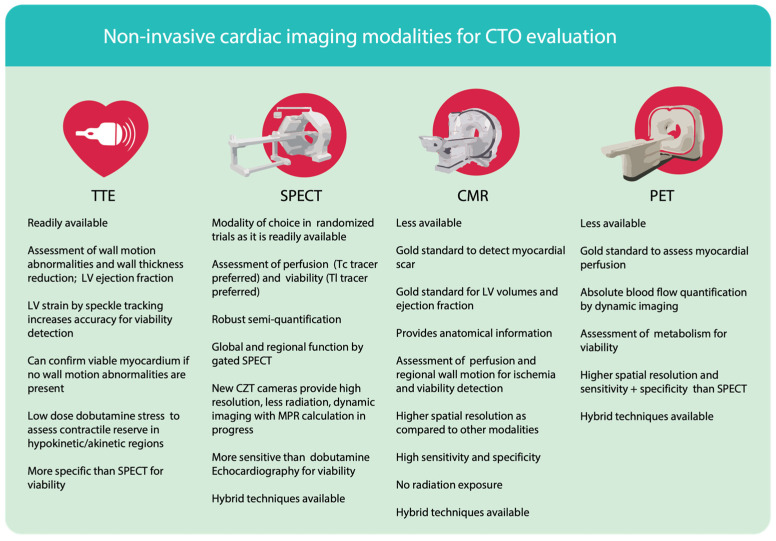
Non-invasive cardiac imaging modalities for CTO evaluation CTO–chronic total occlusion, TTE–transthoracic echocardiography, SPECT–single positron emission computer tomography, CMR–cardiac magnetic resonance, PET–positron emission tomography, LV–left ventricle, Tc–technetium, Tl–thallium.

We reviewed the currently available non-invasive diagnostic tools that can help to set the clinical indication for revascularization in CTO patients and described how they can help properly characterize this patient group by investigating quantitative and qualitative parameters (Figure 1). The availability of diagnostic tools facilitates the investigation of myocardium based on anatomical or functional information and enables a proper patient evaluation. Due to some setbacks in individual imaging methods, hybrid imaging is emerging as an effective approach that increases diagnostic accuracy: PET or SPECT functional information is combined with coronary computed tomography or CMR to facilitate the interpretation of imaging data [44]. To evaluate viability, TTE, SPECT, PET, or CMR could be used. On the other hand, stress protocols using these modalities can help localize and quantify myocardial ischemia.

### 3.1. Transthoracic Echocardiography (TTE)

Resting transthoracic echocardiography (TTE) is a widely available diagnostic method in heart disease and provides an incremental value in characterizing the effects of coronary artery disease on myocardial anatomy and function. As such, it is a fast and efficient way to investigate possible myocardial conditions following a suspected or confirmed coronary artery disease [45,46]. TTE is feasible in patients with an angiographically confirmed CTO, in order to primarily investigate the presence and extent of viability or left ventricular dysfunction. The echocardiographic evaluation of viability includes measurements of wall thickness, characterization of wall shape, and regional wall motion. An end-diastolic wall thinning with less than 5–6 mm usually indicates non-viable myocardial segments. However, wall thinning can be present in viable areas and thus be followed by false-negative interpretations: Shah et al., observed preserved viability in ca. one-fifth of patients with an end-diastolic wall thickness of less than 5.5 mm [47]. On the other hand, wall motion variability may indicate loss of viability: mild hypokinesia mostly indicates a viable region without transmural defects; severe hypokinesia shows high variability and does not exclude viability; akinesia or dyskinesia are somehow more sensitive in indicating a transmural scar, but do not exclude viability [45]. Akinesia is recovered after revascularization, thus indicating viability in the corresponding region [45]. Novel left ventricular strain assessment by two or three-dimensional speckle tracking can also be useful to detect viability and may be somewhat more accurate, as it takes into consideration, among others, longitudinal, radial, and circumferential components of wall motion. The severely reduced strain has indicated transmural scar and preserved strain, on the other hand, has indicated viable myocardial regions [35]. Resting TTE has a low sensitivity to detect myocardial viability in dysfunctional myocardium as it is based on anatomical findings, but it is valuable for the initial exclusion of left ventricular dysfunction. However, these findings in TTE should be interpreted carefully, as in areas of akinesia or hypokinesia viability may be underestimated, as in hibernating myocardium [48].

Dobutamine echocardiography, on the other hand, enables a dynamic assessment of the improvement in contractile function (contractile reserve) in dysfunctional areas and indicates preserved viability. A biphasic response in myocardial wall motion alterations after injection of dobutamine (motion enhancement at low doses and downgrading at high doses) indicates hibernating myocardium and preserved viability [49]. Dobutamine TTE has a high sensitivity of up to 90% for the prediction of improvement in regional or global left ventricular function [50]. Moreover, intravenous contrast injection during dobutamine TTE can help to improve image quality and to assess myocardial perfusion, but has limited clinical use [51]. Stress protocols have for a long time been integrated into standard TTE imaging to assess the impairment of wall motion during pharmacological or physical stress induction [52]. A retrospective temporal study on stress-TTE described a continuous reduction in the predictive value of negative stress tests based on wall motion abnormalities [53].

Echocardiography represents a valuable non-invasive imaging method, but in general it can be limited by reduced image quality in patients with difficult acoustic windows, high interpreter variability, as well as low sensitivity to quantify persistent and stress-induced defects using wall motion assessment.

### 3.2. Single Photon Emission Computer Tomography (SPECT)

**Figure 2:**
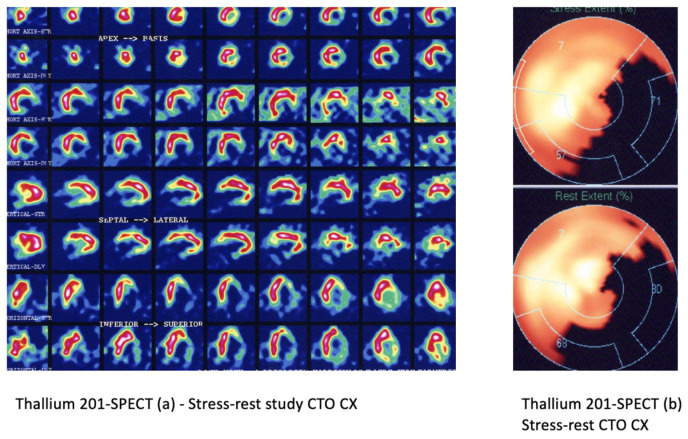
Thallium 201-SPECT study in a patient with a CTO CX as described in the coronary angiography. Short axis views are displayed in row 1–4 and vertical and horizontal long axis views in row 5–8. No significant tracer uptake in stress (row 1,3,5,7) and rest (row 2,4,6,8) acquisitions can be interpreted as lack of viability in the lateral and basal part of inferior myocardial segments (**a**). On the right, a semi-quantification of tracer uptake from the stress (top) and rest (bottom) acquisitions in a polar map (**b**).

Single Photon Emission Computer Tomography (SPECT) detects myocardial perfusion defects by deriving myocardial radiotracer uptake distribution (Figure 2). Diverse protocols include stress, resting and redistribution perfusion assessment. Radionuclides such as thallium-201 and technetium-99 m are used for SPECT imaging. Thallium-201 is flow-dependently allocated in the heart tissue and transported through the intact cell membrane of cardiomyocytes. Resting thallium defects that later reverse in redistribution images (mainly after 4 h) indicate cell integrity and preserved viability. However, redistribution may be delayed and consequently viability may be underestimated [54,55]. Technetium-99 m-labeled radiotracers, sestamibi, and tetrofosmin test the integrity of the mitochondrial membrane. These tracers are less redistributed in the myocardium, which makes them less suitable for viability assessments [56]. On the other hand, they remain feasible for stress-rest assessments of myocardial perfusion but lack hibernation-detecting capability [57].

A classic stress myocardial perfusion imaging protocol is performed after physical or pharmacological stress using vasodilators such as dipyridamole, adenosine, or regadenoson, followed by radiotracer injection. A resting study precedes or follows (optionally only in questionable cases) depending on the selected protocol [58]. Reversible and irreversible stress-rest perfusion defects are quantified using a 17-segment model and a five-point scale, while higher points indicate lower perfusion [59]. Then, summed stress scores (SSS) and summed rest scores (SRS) are subtracted to derive the summed difference scores (SDS) and to subsequently interpret the presence and extent of myocardial scar (SRS) myocardial ischemia (SDS) [60]. Converting SDS into myocardial area percentage is challenging but meaningful, because benefits from revascularization are described relative to myocardial percentages in prior studies [61]. In fact, this semi-quantitative approach is based on a comparison between myocardial segments and has been proven to lack accuracy and underestimate the real extent of myocardial perfusion defects with a sensitivity of ca. 65% [62]. Mostly in patients with balanced multi-vessel disease, SPECT shows a significantly reduced sensitivity [63,64]. Moreover, myocardial perfusion imaging using SPECT can be limited by attenuation artifacts, reduced resolution due to depth dependence and lack of quantitative perfusion measurements [65]. The problem with perfusion quantification has been addressed using new cameras with enhanced resolution and the need for lower tracer dose administration. Currently, new-generation cadmium-zinc-telluride (CZT) cameras enable the acquisition of high-quality SPECT images with the opportunity to use much less radiotracer, thus impressively reducing patient radiation doses. [64] Stress-only SPECT protocols are adapted to the high sensitivity of the camera, thus impressively reducing the radiation doses from 10–15 mSv to ca. 5 mSv on average [66,67]. Due to the high camera sensitivity, short acquisition times with improved image quality in CZT cameras are feasible. Additionally, dynamic acquisition using list-mode recordings enables dynamic evaluation of myocardial perfusion of stress and rest studies and, from that, calculation of myocardial perfusion reserve (MPR) [68,69,70]. Moreover, ECG-gated SPECT with the new CZT cameras can derive functional parameters to characterize left ventricular systolic and diastolic function [71].

### 3.3. Positron Emission Tomography (PET)

**Figure 3:**
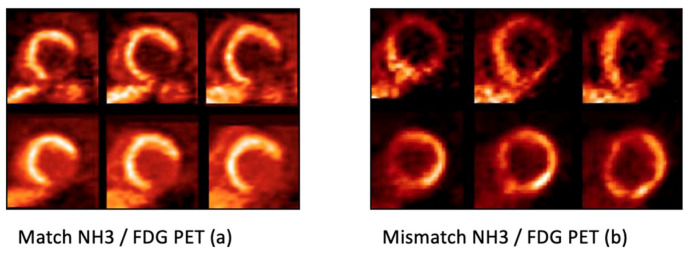
Combined perfusion (performed with N-13 ammonia) and metabolism (performed with F-18 FDG) PET study allows a differentiation between viable and non-viable myocardium. On the left side (**a**) is shown a perfusion/metabolism match in the posterior-lateral segments with equally reduced NH3 (top) and FDG (bottom) uptake reflecting scar in this myocardial region. On the right side (**b**) a perfusion/metabolism mismatch: perfusion (short axis views at the top) and metabolism (short axis views on the bottom) with reduced NH3 uptake and enhanced FDG uptake in the lateral wall. This reflects preserved viability in the hypo-perfused lateral myocardial segments.

Limitations in SPECT perfusion imaging have been outmatched by the use of more precise positron emission tomography (PET) imaging modalities. The concept is to assess myocardial perfusion (using tracer rubidium- 82, O-15 water, or N-13 ammonia) and metabolism (using F-18-FDG) (Figure 3).

FDG is actively transported into the inside of cells by the glucose transporters [72]. In areas with reduced myocardial perfusion a reduced, or lack of, metabolic activity reflects reduced viability. On the other hand, increased glucose metabolism in hypo-perfused areas indicates ischemia and maintained viability [73]. A perfusion-metabolic mismatch has an incremental value in correctly interpreting myocardial viability and identifying ischemia.

Dynamic PET myocardial perfusion assessment enables quantitative information on myocardial blood flow (MBF) and myocardial perfusion reserve (MPR) [62,71,72]. Supported by the enhanced spatial resolution in PET, a qualitative assessment is more accurate and the ability of quantitative measurements provide additional prognostic information in CAD patients [74,75]. By performing similar stress protocols to SPECT imaging, MBF is calculated using two-compartment models and the coronary flow reserve (CFR) is calculated as the ratio of stress MBF and rest MBF [76,77]. Polar mapping can be used to visually border, localize and measure areas with perfusion defects [78]. A correspondent flow status can be then derived from MBF information [41]. In a meta-analysis comparing the two modalities, PET perfusion imaging showed higher sensitivity and similar specificity than SPECT [79].

### 3.4. Cardiac Magnetic Resonance Tomography (CMR)

**Figure 4:**
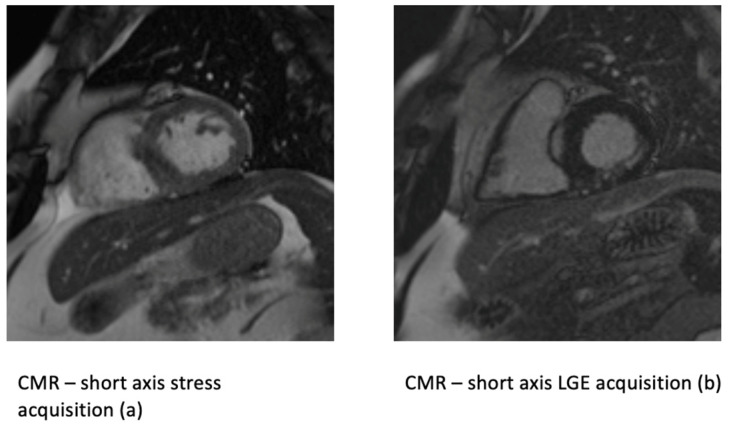
Cardiac magnetic resonance stress acquisition on a short axis view (**a**) with a reduced contrast uptake in posterior-septal segments. (**a**) On the right side (**b**) a late gadolinium enhancement in subendocardial posterior-lateral myocardial segments shows significant preserved viability (<50% trans-murality).

Functional cardiac magnetic resonance tomography (CMR) with late gadolinium enhancement (LGE) is a highly sensitive imaging method for the evaluation of myocardial viability [80]. Gadolinium is a contrast medium that cannot be transported inside the cardiomyocytes and enriches in the interstitial space. In the fibrotic areas with a reduced cardiomyocyte density, delineation of gadolinium-dense spaces can derive spatial information about the existence or extent of scar tissue (Figure 4). The method is highly sensitive, because of the high image resolution and direct measurement of wall thickness, as well as the scar extent in the percentage of total wall thickness. Transmural scar (>50% of total wall thickness) is an indicator of non-viability and is not correlated to any benefits of revascularization. A point scoring system with 0 to 5 points can be used to semi-quantify the quartiles of relative scar extent and give information to interpret the myocardial viability [81]. Viable myocardium in patients with ischemic cardiomyopathy, as assessed by CMR, benefited significantly as compared to medical therapy alone, which underlines the prognostic value and non-inferiority of CMR in viability testing [30]. Furthermore, CMR provides anatomical and functional information similarly to TTE, but with much higher spatial resolution.

While stress-rest SPECT may underestimate viability, the LGE stress and rest CMR can better assess scar, ischemia and hibernating myocardium [82]. Stress perfusion CMR uses the same intravenous contrast admission principle under pharmacological stress with induced hyperemia and rest [83,84]. A 16-segment model on images acquired on the short-axis is then built and ischemic and non-viable areas can be delineated using the scoring system scale in stress and resting conditions. Additionally, the regional wall motion impairment during pharmacological stress and subsequent recovery in resting conditions may indicate ischemia. The novel 3D myocardial perfusion CMR offers better myocardial coverage and accuracy [85,86]. Most importantly, CMR represents a highly accurate diagnostic method with an increased sensitivity of ca. 86% for detection of ischemia in CAD, as compared to the limited sensitivity of ca. 65% in SPECT myocardial perfusion imaging [87]. The CE-MARC and INFORM trials have also underlined the diagnostic accuracy of CMR and equivalence to the gold standard FFR invasive measurement [88]. However, the accuracy of current CMR practice in CTO patients has not been fully validated: patient-tailored protocols for stress CRM could increase the sensitivity for identification of suitable candidates for CTO revascularization. The ongoing CARISMA-CTO study aims to address this issue and finally adapt CMR stress protocols to individual CTO patient characteristics [89].

## 4. Concluding Interpretations and Proposed Workflow

CTO patients are often multimorbid patients with coexisting medical conditions that could alter diagnostics and clinical decisions, which calls for a dedicated patient evaluation in an outpatient clinical setting [90]. For this reason, the interventional community is encouraging careful and comprehensive patient evaluation as well as bilateral physician-patient decision-making before attempting CTO PCI [3]. Patient evaluation for clinical indication should be conducted by physicians who operate CTO PCI themselves or are familiar with the complex character of the condition, since angiographic information should be integrated to non-invasive imaging information in the context of a spatial and quantitative assessment of myocardial wall motion and function, ischemia and viability.

### 4.1. Choice and Interpretations of Non-Invasive Imaging Modalities

**Figure 5:**
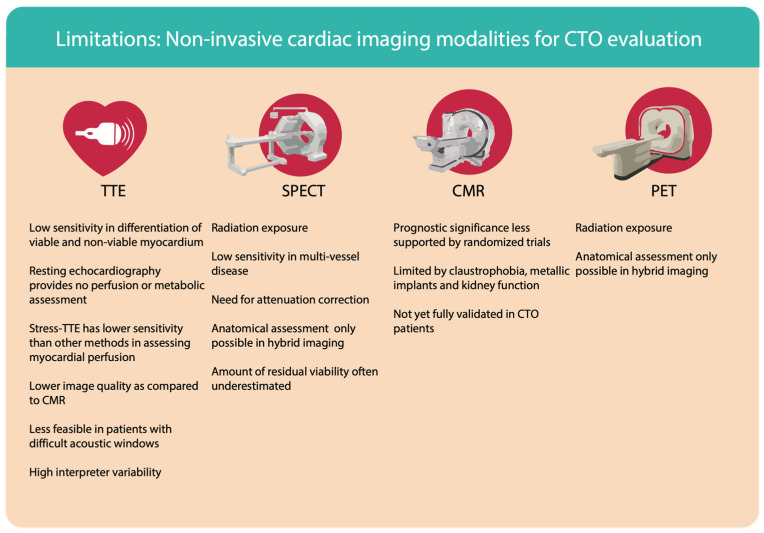
Limitations: Non-invasive cardiac imaging modalities for CTO evaluation: CTO–chronic total occlusion, TTE–transthoracic echocardiography, SPECT–single positron emission computer tomography, CMR–cardiac magnetic resonance, PET–positron emission tomography.

Supported by robust evidence, viability has a pivotal role in predicting short and long-term revascularization benefits. In patients with extended scarring in the CTO region, revascularization benefits are not warranted, while in those with smaller regional defects, the extent of viability could be interpreted along with angiographic information, to finally derive the binary labels of significant viability vs. non-viable CTO region. CMR delivers more accurate information in this context, as it can quantify both the regional extent and thickness of LGE enhancement. SPECT imaging for viability and ischemia is widely accessible and affordable; new CZT cameras and adapted protocols expose patients to less radiation. Thallium-201 SPECT rest-redistribution imaging provides better viability assessment compared to technetium-99 m stress-rest SPECT; in case of doubt, FDG-PET might be performed additionally. In the same manner, ischemia can be underestimated in some cases in the classic segmental semi-quantification (Figure 5). However, given significantly large ischemia, viability, and no confounders to disintegrate SPECT imaging, the indication to undergo CTO PCI remains uncomplicated. On the other hand, PET imaging is a newer method, but less accessible. Due to the possibility of metabolic and perfusion imaging, as well as being a gold standard for myocardial blood flow quantification, PET is a more accurate method for the evaluation of ischemia and viability [91].

Ischemia assessment is a more complex process in CTO patients, which requires careful angiography integration. Due to the development of collaterals, the CTO region and donor vessel are interconnected, thus influencing hemodynamic parameters and myocardial perfusion distribution. The collateral circulation and myocardial “steal” have been long described in the literature and recently tested in FFR studies, implicating two main effects: firstly, collaterals are rarely sufficient to provide enough flow to CTO regions; blood flow is further reduced in hyperemia due to coronary steal [92]. Secondly, collateral “leak” may cause impaired flow in the distal donor vessel area during vasodilator stress induction, hence reducing remote myocardial perfusion. Indeed, recent studies have reported an increased remote myocardial perfusion after CTO PCI [93]. This may result in surprising findings in pre-interventional ischemia testing.

### 4.2. Proposed Workflow

Symptom improvement is the primary indication for CTO PCI with the strongest evidence to date. For this reason, ischemic symptoms and the will to accept the peri-interventional risks for improvement of life quality should stay on the plateau of the information cluster when conducting the pre-interventional evaluation. Secondly, the prognostic benefits of CTO PCI can be evaluated and taken into consideration, as stated in the European guidelines (CTO in left anterior descending or left main coronary artery, multi-vessel disease, reduced ejection fraction—Figure 5), especially in the case of expert operators, with high success and low complication rates.

**Figure 6:**
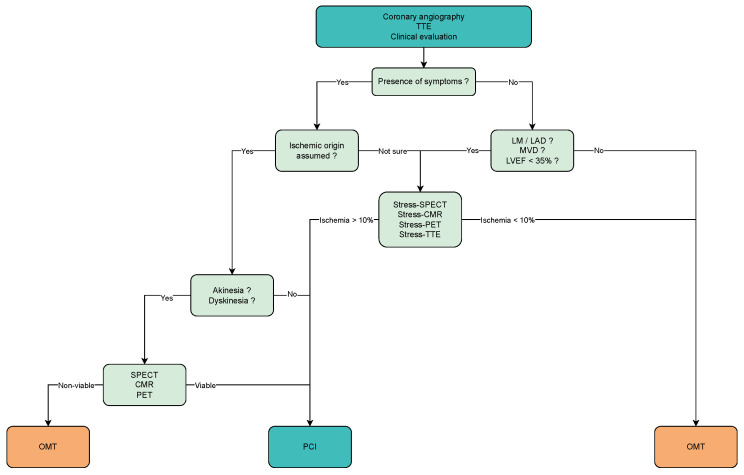
Workflow for clinical indication of CTO PCI, TTE—trans-thoracic echocardiography; LM—left main coronary artery; LAD—left anterior descending coronary artery; LVEF—left ventricular ejection fraction; SPECT—single photon emission computer tomography; CMR—cardiac magnetic resonance; PET—positron emission tomography; OMT—optimal medical therapy; PCI—percutaneous coronary intervention.

Our proposed systematic workflow is presented in Figure 6. This workflow should be interpreted as a suggestion and an attempt to partially integrate the limited evidence in clinical practice. The process of clinical pre-interventional assessment in intervention-eligible CTO patients can begin with objective symptom assessment and investigation of other factors causing the symptoms, as well as comorbidities and physical capacity. Angina or dyspnoea scoring systems as well as physical performance assessment using treadmill or bicycle ergometry or the more simplified 6-min walking test can be useful to document the presence of symptoms [94]. Structural abnormalities of the heart, including wall motion and ventricular function can be assessed using a resting TTE. If ventricular dysfunction is lacking, viability can be assumed; otherwise, further viability testing may be useful. This information in combination with cardiac biomarkers can be useful for an eventual initial optimization of medical therapy. If unsure about the persisting symptom origin or mild symptoms indicative of the presence of ischemia, ischemia testing could be then performed. SPECT imaging is a feasible technique in the case of 1-vessel disease and uncomplicated angiographic anatomy. If viability testing is explicitly needed, SPECT imaging can be carefully interpreted to conclude if the viability presence is plausible. In more complex anatomic cases or multivessel disease and no contraindications to CMR, stress-CMR or PET can be very accurate alternatives with somewhat lower availability. If the severity and ischemic origin of symptoms is assumed, stress protocols can also be avoided. In asymptomatic patients with angiographic small CTO vessels not suitable for intervention, the situation can be explained to the patient and conservative management of CAD can be followed, to avoid unnecessary testing.

In conclusion, in the context of the lack of established patient evaluation algorithms including recommendations of specified cardiac imaging methods, this review has to be read as a suggestion according to the literature and the experience of the authors. Indeed, the evaluation process workflow is currently left to the physician’s discretion. However, patient evaluation should be conducted by physicians who are familiar with imaging techniques, complex coronary angiography and CTO PCI.

## Figures and Tables

**Table 1:** Randomized studies comparing CTO PCI with OMT [4,5,6,7,8] CTO PCI—percutaneous coronary intervention of chronic total occlusion, OMT—optimal medical therapy, MACE—major adverse cardiac events, QOL—quality of life, LVEF—left ventricular ejection fraction, CMR—cardiac magnetic resonance, LVEDV—left ventricular end-diastolic volume, SWT—segmental wall thickening, (I)—primary endpoint, (II)—secondary endpoint, (subgroup)–results derived from subgroup analysis; **—reporting of viability or ischemia data.

Study	Time	Number of Patients	Success Rate	Follow-Up (Median)	Findings
DECISION-CTO	2010–2016	834 (1:1)	90.6%	4 years	- No difference in MACE occurence (I) - Better QOL in CTO PCI group (II) - ** no data on ischemia and viability detection
EURO-CTO	2012–2015	396 (2:1)	86.6%	1 year	- Better QOL and Angina reduction in CTO PCI group (I) - No difference in MACE occurence (II) - ** Ischemia PCI arm 65%, Viability PCI arm 86%
EXPLORE	2007–2015	304 (1:1)	77%	4 months	- No benefit in LVEF (CMR) nor in LVEDV (I) - LAD CTO PCI had higher LVEF (subgroup) - No benefit in terms of MACE (II) - ** no data on ischemia and viability detection
REVASC	2007–2015	205 (1:1)	86% at first attempt (99% overall)	1 year	- No benefit in terms of SWT, regional and global LVEF (CMR) (I) - CTO PCI had less MACE driven by repeat PCI (II) - Single vessel disease CTO patients benefited from PCI in terms of SWT (subgroup) - ** no data in ischemia and viability detection
IMPACTOR (RCA CTO)	2010–2014	94 (1:1)	83%	1 year	- CTO PCI group had a significant MIB decrease compared to OMT - Better QOL in the CTO PCI group - No difference in terms of MACE - ** myocardial ischemic burden documented, no data on viability

**Table 2:** Guideline recommendations for CTO PCI [16,17].

Gudielines	Class of Recommendation	Level of Evidence	Recommendation
European 2018	II-a	B	“Percutaneous revascularization of CTOs should be considered in patients with angina resistant to medical therapy or with a large area of documented ischaemia in the territory of the occluded vessel”
American 2021	II-b	B	“In patients with suitable anatomy who have refractory angina on medical therapy, after treatment of non-CTO lesions, the benefit of PCI of a CTO to improve symptoms is uncertain”
